# Validation of the Vasoactive-Ventilation-Renal Score for Neonatal Heart Surgery

**DOI:** 10.7759/cureus.15110

**Published:** 2021-05-19

**Authors:** Adil Umut Zubarioglu, Özgür Yıldırım, Cenap Zeybek, İsmail Balaban, Volkan Yazıcıoglu, Bahruz Aliyev

**Affiliations:** 1 Neonatology, Yeni Yuzyıl University, Istanbul, TUR; 2 Cardiovascular Surgery, Yeni Yuzyıl University, Istanbul, TUR; 3 Pediatric Cardiology, Yeni Yuzyıl University, Istanbul, TUR

**Keywords:** neonate, postoperative care, congenital heart disease, cardiac intensive care, vvr score

## Abstract

Objectives: We aimed to validate the vasoactive-ventilation-renal (VVR) score and to compare it with other indices as a predictor of outcome in neonates recovering from surgery for critical congenital heart disease. We also sought to determine the optimal time at which the VVR score should be measured.

Methods: We retrospectively reviewed neonates recovering from cardiac surgery between July 2017 and June 2020. The VVR score was calculated at admission, 24, 48, and 72 hours postoperatively. Max values, defined as the highest of the four scores, were also recorded. The main end result of interest was a composite outcome which included prolonged intensive care unit stay and mortality. Receiver operating characteristic curves were generated, and areas under the curve with 95% confidence intervals were calculated for all time points. Multivariable logistic regression modeling was also performed.

Results: We reviewed 73 neonates and 21 of them showed composite outcomes. The area under the curve value for VVR score as a predictor of composite outcome was greatest at postoperative 72-hour max (AUC= 0.967; 95% confidence interval, (0.927-1). On multivariable regression analysis, the VVR max 72 hours remained a strong independent predictor of prolonged ICU stay and mortality (odds ratio, 1.452; 95% confidence interval, 1.036-2.035).

Conclusions: We validated the utility of the VVR score in neonatal cardiac surgery for critical congenital heart disease. The VVR follow-up in postoperative 72 hours is superior to other indices and especially the maximum VVR value is a potentially powerful clinical tool to predict ICU stay and mortality.

## Introduction

Newborns operated for critical congenital heart disease (CHD) have a high risk of postoperative mortality and morbidity [1-4]. These patients have a long recovery period that requires serious hemodynamic and respiratory support in the postoperative period [2,5]. While the potential adverse outcomes that may be seen during this period are well defined, it is difficult to determine which patients are at high risk for poor prognosis due to the heterogeneity of the anatomy and pathophysiology. For this reason, there is a need for indices that can help to provide the necessary and adequate support by determining the severity of not just cardiopulmonary dysfunction using clinical and laboratory data in the early postoperative period.

For this purpose, it has been shown that the vasoactive inotrope score (VIS) and serum lactate levels, which are used recently in clinical practice, are correlated with prognosis [6-9]. VIS is based on the measurement of pharmacological agent support required to provide adequate hemodynamic stability in the postoperative period, while serum lactate is the indicator of end-organ perfusion.

However, newborns undergoing cardiac surgery often have multiorgan dysfunction and especially pulmonary and renal systems are affected [10,11]. Neither VIS nor serum lactate show these system functions.

In order to overcome this limitation of serum lactate and VIS, Miletic et al. introduced the vasoactive-ventilation-renal (VVR) score in 2015 and used markers of the cardiovascular, pulmonary, and renal systems, which are the most frequently affected systems in CHD surgery [12]. In the following years, the VVR score was found to be a strong predictor in determining the prognosis and gave better results than other indices in a limited number of studies performed by comparing the values at different postoperative periods in various pediatric age groups and heterogeneous disease population [12-14]. However, there is limited data about VVR score in the newborn population, which is the riskiest population in terms of age group and disease complexity in congenital heart surgery. Especially, postoperative timing and cut-off values of VVR score for efficient prediction of prognosis in neonates are uncertain.

In the most recent multicenter retrospective study involving only newborns, it was found that the postoperative twelfth-hour VVR score was more effective in predicting the prolonged mechanical ventilation (MV) duration compared to other indices, Society of Thoracic Surgeons - European Association for Cardio-Thoracic Surgery Congenital Heart Surgery (STAT) Mortality Categories and cardiopulmonary bypass (CPB) duration [15].

However, in the vulnerable neonatal populations, the possibility to have temporary cardiovascular, pulmonary, and renal dysfunction in the early postoperative period is higher. Therefore, it would be rational to follow up these patients with VVR scores for a longer period not to miss out on the sustained dysfunction which is more likely to influence the outcome.

The aim of our study is to evaluate the potential of predicting prolonged intensive care unit (ICU) stay and/or mortality by calculating the VVR score for 72 hours postoperatively, and to compare it with other indices and, if possible, to find an appropriate cut-off value and to provide an idea for future studies.

This article was previously posted to the Authorea preprint server on October 29, 2020 (https://authorea.com/users/371116/articles/489571-validation-of-the-vasoactive-ventilation-renal-score-as-a-predictor-of-prolonged-intensive-care-unit-stay-and-mortality-after-critical-congenital-heart-surgery-in-neonates).

## Materials and methods

Design and setting

The study was a retrospective single-center study conducted in a three-year period between July 2017 and June 2020. The study was approved by the institutional ethical committee (file number:1621). Informed written consent were obtained from the parents of all participants for surgical and intensive care procedures.

Study population

All neonates, defined as less than or equal to 28-days-old, who underwent pediatric cardiac surgery (with or without CPB) were reviewed.

Exclusion criteria were as follows: (i) premature babies who underwent ligation of patent ductus arteriosus (PDA); (ii) cases that placed on extracorporeal membrane oxygenation (ECMO) support in the operating room or those requiring ECMO support within the first 72 hours postoperatively were excluded from the study.

Data collection

Data were collected via medical record review. Demographic and preoperative data collection included sex, birth weight, gestational week, being premature (<37 gestational weeks), being low birth weight (<2500 g), antenatal diagnosis status, postnatal admission day, underlying cardiovascular diagnoses, presence of noncardiac anomalies, need for MV and inotropic support at admission, need for MV and inotropic support at operation day, preoperative sepsis and need of dialysis, and postnatal day of surgery were recorded.

Perioperative data collection included surgical procedure performed, STAT Mortality Category [16], use and duration of CPB and aortic cross-clamping, use of deep hypothermic arrest, and antegrade cerebral perfusion were recorded.

Postoperative data collection included the need for ECMO, need for inhaled nitric oxide therapy, presence of postoperative open sternotomy, the occurrence of arrhythmias, postoperative MV and inotropic duration, duration of ICU stay, and mortality were recorded. 

We recorded MV variables, arterial blood gas and lactate measurements, and doses of inotropic and vasopressor medications at four postoperative time points: ICU admission, 24 hours, 48 hours, and 72 hours after ICU arrival. We also recorded preoperative serum creatinine and daily serum creatinine measurements obtained within the first 72 postoperative hours.

Derivation of the VVR

VVR scores were calculated as follows: VI (Ventilation Index) + VIS + ΔCr.

VIS = dopamine dose [μg/kg/min] + dobutamine dose [μg/kg/min] + 100 × epinephrine dose [μg/kg/min] + 10 × milrinone dose [μg/kg/min] + 10,000 × vasopressin dose [U/kg/min] + 100 × norepinephrine dose [μg/kg/min]).

VI = ([ventilator respiratory rate] × [peak inspiratory pressure - positive end-expiratory pressure] × PaCO_2_))/1,000).

ΔCr = change in creatinine from baseline × 10.

VIS, VI, and ΔCr were calculated at each postoperative time point. VI and VIS were recorded as zero for patients not receiving mechanical ventilation or inotropic support, respectively. Baseline preoperative serum creatinine was subtracted from each postoperative serum creatinine measurement. For patients whose postoperative serum creatinine measurements were less than or equal to baseline, ΔCr was assumed to be equal to 0.

Outcome

Our main outcome variable was postoperative prolonged ICU duration and/or mortality which we expressed as a composite outcome. We defined prolonged ICU duration as the upper 25% of the study population according to the hospital stay.

Statistical analysis

All statistical analyses were performed using IBM SPSS version 23 (IBM Corp., Armonk, NY). Shapiro-Wilk tests were used to determine normal distribution. The main outcome of interest was the composite outcome (postoperative prolonged ICU stay and/or mortality). The study group was dichotomized as upper 25th percentile of ICU stay and/or mortality (defined as having composite outcome) versus lower 75th percentile for ICU stay. Bivariate comparisons were performed for demographic and perioperative characteristics of patients with and without composite outcome using Chi-square or Fisher exact test for categorical variables. In the comparison of quantitative variables according to paired groups, an independent two-sample t-test was used for normally distributed data and the Mann-Whitney U test was used for non-normally distributed data. Repeated analysis of variance was used to compare normally distributed data over three or more times, and the Friedman test was used to compare non-normally distributed data. Variables that attained a bivariate significance of 0.2 or less were considered for inclusion in a multivariable logistic regression model. Receiver operating curve (ROC) analysis was used to determine cut-off values for VIS, VI, VVR, VVR_max72_, serum lactate, and CPB duration values according to the composite outcome. Analysis results are represented as mean ± standard deviation (SD) and median (25th-75th percentile) for quantitative data. It is presented as frequency (percentage) for categorical data. The significance level was taken as p<0.05.

## Results

The medical files of 95 newborns who were operated on in the first 28 days of life during the study period were examined. Sixteen of these patients were preterm babies who had bedside PDA ligation. Six patients were excluded from the study because they were placed on ECMO support in the operation room. As a result, the study group consisted of 73 patients. The anatomical diagnoses of the patients included in the study and the surgical procedures performed are summarized in Table 1. The most common anatomical diagnoses were hypoplastic left heart syndrome (26%), transposition of great arteries (16.4%), aortic arch hypoplasia (12.3%), and coarctation of the aorta (12.3%).

**Table 1:** Type of congenital heart diseases and primary surgical procedures in neonates enrolled in the study. IVS: intact ventricular septum, VSD: ventricular septal defect, AVSD: atrioventricular septal defect, DORV: double outlet right ventricle.

	Without composite outcome	With composite outcome	Total (n=73)
Anatomic diagnosis, n (%)			
Coarctation of aorta	9 (17.3)	0 (0)	9 (12.3)
Pulmonary atresia + IVS	0 (0)	1 (4.8)	1 (1.4)
Pulmonary atresia + VSD	3 (5.8)	0 (0)	3 (4.1)
Coarctation of aorta + VSD	2 (3.8)	0 (0)	2 (2.7)
Aortic arch hypoplasia	8 (15.4)	1 (4.8)	9 (12.3)
Aortic interruption + VSD	4 (7.7)	2 (9.5)	6 (8.2)
Transposition of great arteries	10 (19.2)	2 (9.5)	12 (16.4)
DORV	1 (1.9)	0 (0)	1 (1.4)
Total anomalous pulmonary venous return	3 (5.8)	2 (9.5)	5 (6.8)
Hypoplastic left heart syndrome	6 (11.5)	13 (61.9)	19 (26)
Pulmonary atresia + DORV + AVSD	1 (1.9)	0 (0)	1 (1.4)
Tricuspid atresia	4 (7.7)	0 (0)	4 (5.5)
Truncus arteriosus	1 (1.9)	0 (0)	1 (1.4)
Primary surgical procedure, n (%)			
Norwood stage 1	5 (9.6)	11 (52.4)	16 (21.9)
Coarctation repair, end to end	6 (11.5)	0 (0)	6 (8.2)
Coarctation repair, end to end, extended	3 (5.8)	0 (0)	3 (4.1)
Coarctation repair + VSD repair	1 (1.9)	0 (0)	1 (1.4)
Aortic arch repair	8 (15.4)	1 (4.8)	9 (12.3)
Arterial switch procedure + VSD repair	9 (17.3)	2 (9.5)	11 (15.1)
Interrupted aortic arch repair	4 (7.7)	2 (9.5)	6 (8.2)
Truncus arteriosus repair	1 (1.9)	0 (0)	1 (1.4)
Pulmonary artery banding	6 (11.5)	1 (4.8)	7 (9.6)
Valvuloplasty, pulmonic	0 (0)	1 (4.8)	1 (1.4)
Pulmonary atresia-VSD repair	2 (3.8)	0 (0)	2 (2.7)
Hybrid approach stage 1	0 (0)	1 (4.8)	1 (1.4)
Total anomalous pulmonary venous return repair	3 (5.8)	2 (9.5)	5 (6.8)
Systemic to pulmonary artery shunt	3 (5.8)	0 (0)	3 (4.1)
Modified Blalock-Taussig shunt	1 (1.9)	0 (0)	1 (1.4)

The mean gestational week and birth weight of the study group was 38.6 ± 1.6 weeks and 3179 ± 528 g, respectively. Over 72.6% of the cases were male, 12.3% were premature, and 13.7% had an additional non-cardiac anomaly. The antenatal diagnosis rate was 28.8%. 38.4% of the cases had single ventricle anatomy and 82.2% were STAT category 4 or 5. None of the cohorts needed ECMO support in the first 72 hours postoperatively.

The postoperative prolonged ICU stay of the cohort was defined as 29 days or longer. Eight cases died in the postoperative period. Two of these cases died before 29 days (15th and 20th days). Thus, 21 cases met the composite outcome criterion, which is the main outcome of our study. The study population was divided into two according to the composite outcome, and bivariate analysis of demographic, anthropometric, and perioperative features of cases with and without composite outcome are presented in Table 2. Of the cases that result with the composite outcome; the rate of MV requirement at admission and on the operation day, the rate of having single ventricle anatomy, the rate of being in the STAT 5 category, the rate and duration of CPB, the rate of applying aortic cross-clamp, the rate of leaving the operation with the open sternum, postoperative MV need, inotrope need, and ICU stay was significantly higher.

**Table 2:** Demographic, anthropometric, and perioperative data. SD: standard deviation, MV: mechanical ventilation, STAT: Society of Thoracic Surgeons-European Association for Cardio-Thoracic Surgery, CPB: cardiopulmonary bypass, DHCA: deep hypothermic circulatory arrest, ACP: antegrade cerebral perfusion, NO: nitric oxide ICU: intensive care unit.

Variables	All patients (n=73)	With composite outcome (n=21)	Without composite outcome (n=52)	p-Value
Gestational weeks (week), mean ± SD	38.6 ± 1.4	38.9 ± 1.1	38.4 ± 1.6	0.139
Weight (g), mean ± SD	3179.1 ± 528.2	3136.1 ± 416.6	3196.5 ± 569.9	0.662
Gender (male), n (%)	53 (72.6)	17 (81)	36 (69.2)	0.309
Prematurity (<37 weeks), n (%)	9 (12.3)	1 (4.8)	8 (15.4)	0.432
Noncardiac anomalies, n (%)	10 (13.7)	2 (9.5)	8 (15.4)	0.714
Antenatal diagnosis, n (%)	21 (28.8)	5 (23.8)	16 (30.8)	0.552
Admission day, median (25th–75th percentile)	4 (2–9)	3 (2–6)	4.5 (2–9.5)	0.018
MV needs at admission, n (%)	31 (42.4)	13 (61.9)	18 (34.6)	0.048
Inotrope needs at admission, n (%)	25 (34.2)	8 (38.1)	17 (32.7)	0.660
MV needs at the day of surgery, n (%)	32 (43.8)	15 (71.4)	17 (32.7)	0.003
Inotrope needs at the day of surgery, n (%)	30 (41.1)	9 (42.9)	21 (40.4)	0.846
Preoperative sepsis, n (%)	3 (5.8)	0 (0)	3 (4.1)	0.552
Age at surgery (day), median (25th–75th percentile)	11 (7–19)	7 (5–10)	13 (8–21.5)	<0.001
Single ventricle anatomy, n (%)	28 (38.4)	14 (66.7)	14 (26.9)	0.002
STAT* mortality category, n (%)				0.001
STAT Category 1	6 (8.2)	0 (0)	6 (11.5)	
STAT Category 2	4 (5.4)	1 (4.7)	3 (5.7)	
STAT Category 3	3 (4.1)	0 (0)	3 (5.7)	
STAT Category 4	44 (60.3)	9 (42.9)	35 (67.3)	
STAT Category 5	16 (21.9)	11 (52.4)	5 (9.6)	
STAT Categories 4&5, n (%)	60 (82.2)	20 (95.2)	40 (76.9)	0.092
Cases undergoing CPB, n (%)	59 (80.8)	21 (100)	38 (73.1)	0.007
CPB duration (min), median (25th–75th percentile)	128 (98–164)	163 (128–198)	121.5 (89–147)	0.009
Aortic cross-clamp application, n (%)	57 (78.1)	20 (95.2)	37 (71.2)	0.029
Aortic cross-clamp duration (min), median (25th–75th percentile)	80 (61–110)	100.5 (74.5–119)	75 (60–95)	0.059
DHCA, n (%)	45 (61.6)	16 (76.2)	29 (55.8)	0.104
ACP, n (%)	43 (58.9)	16 (76.2)	25 (48.1)	0.056
Postoperative open sternum, n (%)	31 (42.5)	19 (90.5)	12 (23.1)	<0.001
Postoperative inhaled NO use, n (%)	2 (2.7)	1 (4.8)	1 (1.9)	0.495
Postoperative arrhythmias, n (%)	12 (16.4)	5 (23.8)	7 (13.5)	0.308
Postoperative MV duration (day), mean ± SD	9.4 ± 13.1	23.2 ± 17.5	3.8 ± 3.1	<0.001
Postoperative inotrope duration (day), mean ± SD	15.3 ± 14.1	31.1 ± 16.1	8.8 ± 5.5	<0.001
Postoperative ICU stay (day), mean ± SD	24.6 ± 15.6	42.5 ± 17.3	17.4 ± 5.8	<0.001

ROC analysis of serum lactate, VIS, VI, and VVR scores was performed, and area under curve (AUC) values, cutoff values, sensitivity, and specificity data at all measurement points are presented in Table 3. VVR performed well and had a greater AUC than the corresponding VIS, VI, and serum lactate at each measurement point. Since it reflects the data of all cases and the AUC values were the highest, bivariate analysis was performed in terms of the composite outcome of the 72-hour highest values of all cases and presented in Table 4. All score indices and serum lactate levels were found to be significantly higher in the composite outcome group.

**Table 3:** Receiver operating characteristic analysis of postoperative predictive variables and the composite outcome. AUC: area under the curve, VIS: Vasoactive Inotrope Score, VI: Ventilation Index, VVR: Vasoactive Ventilation Renal Score, CPB: cardiopulmonary bypass.

	AUC (95% CI)	Cut-off point	Sensitivity	Specificity
Admission				
Lactate	0.847 (0.742–0.952)	≥4.83	0.762	0.733
VIS	0.818 (0.705–0.931)	≥16.5	0.571	0.900
VI	0.713 (0.568–0.859)	≥26.5	0.667	0.567
VVR	0.854 (0.754–0.954)	≥42.5	0.714	0.733
24-hour				
Lactate	0.863 (0.763–0.962)	≥4.67	0.810	0.800
VIS	0.828 (0.718–0.938)	≥16.5	0.619	0.867
VI	0.843 (0.732–0.954)	≥28.5	0.762	0.767
VVR	0.921 (0.845–0.998)	≥45.5	0.857	0.900
48-hour				
Lactate	0.801 (0.677–0.925)	≥3.23	0.762	0.733
VIS	0.864 (0.765–0.964)	≥16.5	0.714	0.900
VI	0.827 (0.714–0.94)	≥26.5	0.714	0.733
VVR	0.901 (0.812–0.996)	≥42	0.857	0.833
72-hour				
Lactate	0.876 (0.780–0.972)	≥3.11	0.810	0.833
VIS	0.89 (0.799–0.98)	≥14.75	0.857	0.767
VI	0.878 (0.785–0.97)	≥25.75	0.810	0.800
VVR	0.924 (0.851–0.996)	≥39.5	0.952	0.833
Peak at 72-hour				
Lactate	0.911 (0.835–0.986)	≥6.03	0.857	0.868
VIS	0.886 (0.802–0.975)	≥16.5	0.714	0.895
VI	0.922 (0.856–0.987)	≥29.5	0.905	0.816
VVR	0.967 (0.927–1)	≥46.5	0.905	0.868
CPB duration	0.718 (0.573–0.863)	≥148.5	0.667	0.763

**Table 4:** Bivariate analysis of postoperative scoring indices max values comparing patients with and without composite outcome. All data represented as median (IQR). VIS: Vasoactive Inotrope Score, VI: Ventilation Index, VVR: Vasoactive Ventilation Renal Score, IQR: interquartile range.

Variable	Without composite outcome (n=52)	With composite outcome (n=21)	p-Value
Lactate max	4.09 (3.3–5)	7 (6.25–7.56)	<0.001
VIS max	12 (10–15)	18 (15–20)	<0.001
VI max	26 (22–29)	35 (32–39)	<0.001
VVR max	37.5 (32–43.5)	53 (49–57)	<0.001

All variables in Tables 2 and 4 with a p-value less than 0.2 were considered for the multivariable model. On multivariable regression analysis, the VVR 72 hour max remained a strong independent predictor of the composite outcome. Specifically, with each increase of 1 in a patient’s VVR_72max_ score, the odds of a composite outcome increased by 45% (odds ratio, 1.45; 95% CI, 1.03-2.03). The remaining variables that were significant on bivariate analysis were not significant on multivariable analysis and did not appreciably affect the model. The best multivariable model for composite outcome including VVR 72 hour max, lactate 72-hour max, and single ventricle physiology is presented in Table 5.

**Table 5:** Multivariate logistic regression analysis for predictors of the composite outcome. SE: standard error, OR: odds ratio, VVR: Vasoactive Ventilation Renal score.

Parameter	Beta	SE	p-Value	OR (95% CI)
Single-ventricle anatomy	−0.444	0.258	0.086	0.642 (0.387–1.064)
VVR 72-hour max	0.373	0.172	0.030	1.452 (1.036–2.035)
Lactate 72-hour max	1.082	0.636	0.089	2.95 (0.848–10.26)
Intercept	−20.267	7.413	0.006	

To simplify the interpretation and use of the VVR_72max_, we dichotomized the variable into high and low. A VVR_72max_ cutoff value of 46.5 was chosen to maximize total accuracy and minimize weighted error ratios, and correctly classified 90% of cases. On multivariable logistic regression analysis, a high VVR_72max_ remained strongly associated with the composite outcome (odds ratio, 23.4; 95% CI, 3.2-170.3).

## Discussion

We have validated the VVR score as a multiorgan system severity of illness index for neonates recovering from surgery for critical congenital heart disease. We demonstrated that the VVR strongly predicts prolonged postoperative ICU stay and/or mortality (composite outcome) more so than VIS, VI, and serum lactate.

In a limited number of prior studies, the VVR calculated at 48 hours (12,13) and at 12 hours [14,15] was found superior to admission and peak VVR measurements. We also found that admission measurement was performed poorly according to other measurement points. In fact, we expected these results, because vasoactive and ventilator support on admission is related to dynamic processes after cessation of CPB rather than organ dysfunction and illness severity. In addition, mostly support at admission may be the highest degree which will negatively affect the strength of VVR measurements. For the neonatal period, this transitional postoperative period lasts much longer than older pediatric age groups with higher vasoactive and ventilatory support. That is why we calculated VVR scores for 72 hours which was longer among prior studies. We demonstrated that the VVR calculated at postoperative 72 hours in neonates can predict ICU stay and mortality superior to other measurement points.

In order to include all cases in the analysis, we compared the highest measurements (VVR_72max_) of each case in the first 72 hours with the maximum values of other indices, and unlike other studies, we reached the highest AUC value and highest accuracy in prediction of results. Specifically, if the postoperative VVR_72max_ of a neonate is 46.5 and above, this case was found to be 23 times riskier in terms of prolonged ICU stay and mortality.

The most important advantage of the VVR score compared to other indices is that it includes patients who are hemodynamically stable and do not need much support, but who have postoperative lung and kidney damage. Making these calculations with routine laboratory data in clinical practice and a simple calculation at the bedside makes it easier to use.

However, the main problems for widespread clinical use in a heterogeneous disease group as congenital heart diseases are the uncertainties about which age group, at what time postoperatively, and what the cutoff values should be. With more studies to be done in the future, the patient's age group and disease-specific cut-off values will be determined.

In the retrospective study of Miletic et al. in which the VVR score was first introduced and its performance was tested; patients under one year of age and without CPB were included in the study, and patients with single ventricle anatomy were excluded. In this study, postoperative 48th-hour VVR score predicted both prolonged MV duration (defined as upper 25 of cohort) and hospital stay better than VIS and serum lactate level. The cutoff value in this study was found to be 22.5 [12].

In the next prospective study of Miletic et al., the patient population was expanded as under 18 years of age and those entering CPB. The VVR index at postop 48th hour gave better results than all measurements, and the cutoff value was found to be 13 [13].

Scherer et al. included pediatric and adult congenital heart patients in their study, and within 48-hour postoperative measurements; it was stated that the predictivity of the 12th-hour VVR score was the most effective, and the cutoff value was determined as 25 [14].

In a recent multi-center study involving only newborns, like our study, score calculations were made in the postoperative 12-hour period, and the VVR score was found to be superior in determining prolonged MV duration (determined as > 96 hours in this study) compared to VIS, VI, and serum lactate. In this study, the cutoff value was expressed as 35 [15].

As it can be seen, as the study age group descends into complex patient groups such as the neonatal period, the cutoff values increase, but the VVR score maintains its strong predictivity compared to other indices.

In our study, 82% of the patient group was in STAT mortality categories 4 and 5 and 80% had undergone CPB. Also, the rate of antenatal diagnosis was as low as 28.8%, so an important part of the study group applied to the hospital with cardiopulmonary decompensation, and 44% required preoperative MV. So our cohort consists of more severe and complex cases according to the literature. We think this is the reason why we found the VVR score cutoff value to be higher.

Although VVR is a powerful marker in determining outcomes like postoperative ICU stay and mortality, there are many additional factors that affect outcomes, especially in neonates. For example, even if our study could not be demonstrated due to the limited number of cases, airway anomalies and gastrointestinal anomalies can prolong hospitalization and cause deaths even if the cardiac operation is successful. In addition, cases without antenatal diagnosis or with delays in postnatal diagnosis and treatment admit to the hospital with cardiopulmonary decompensation, and the multiorgan injuries that occur because of this, affect the postoperative process negatively. The next logical step will be increasing the predictivity of the VVR score by adding well-defined preoperative variables and other organ system markers in future studies.

Longer scoring time in vulnerable populations like the neonatal period will enable us to predict persistent dysfunction leading to poor prognosis rather than acute dysfunction in the postoperative transition period. As our study shows, 72-hour values have the best predictivity after the maximum value in neonatal cardiac surgery. With multi-center and larger heterogeneous patient cohorts, specific cutoff values should be determined according to anatomical diagnosis, age group, surgical procedure, and postoperative measurement time.

Study limitations

Our study has been designed retrospectively and includes only newborn cases. Therefore, multicenter and comprehensive prospective studies are needed regarding the applicability of the VVR score to all congenital heart diseases. We acknowledge that the VVR score cannot reliably be calculated for patients requiring ECMO support and peritoneal dialysis. Studies are needed which focus on this subgroup of patients to identify risk factors for poor outcomes.

## Conclusions

We validated the utility of the VVR score in neonatal cardiac surgery for critical congenital heart disease. The VVR follow-up in postoperative 72 hours is superior to other indices, and especially, the maximum VVR value is a potentially powerful clinical tool to predict ICU stay and mortality. Neonates with a peak VVR greater than or equal to 46.5 within the first 72 postoperative hours should be considered at increased risk for poor prognosis.
